# Inflammatory Mediators in Autoimmunity and Systemic Autoimmune Diseases

**DOI:** 10.1155/2015/878712

**Published:** 2015-08-05

**Authors:** Britt Nakken, György Nagy, Peter C. Huszthy, Even Fossum, Yrjö Konttinen, Peter Szodoray

**Affiliations:** ^1^Department of Immunology, Oslo University Hospital Rikshospitalet and University of Oslo, Oslo, Norway; ^2^Department of Genetics, Cell and Immunobiology, Semmelweis University, Budapest, Hungary; ^3^K. G. Jebsen Center for Influenza Vaccine Research, Institute of Immunology, University of Oslo and Oslo University Hospital, Oslo, Norway; ^4^Department of Medicine, Institute of Clinical Medicine, Helsinki University Central Hospital and ORTON Orthopaedic Hospital of the Invalid Foundation, Helsinki, Finland

Autoimmune processes can be found physiologically, as a natural phenomenon in humans. A wide array of surveillance mechanisms, cells with regulatory properties, and tolerance mechanisms exists to control and decelerate autoimmunity and to avoid the development of autoimmune diseases. However, when not properly controlled, the intricate interplay of genetic, environmental, and immunological factors leads to the development of these debilitating diseases. The immune system has multiple levels of negative feedback mechanisms that dampen immune responses and counteract the establishment of chronic and destructive immunity. Exponentially growing knowledge on inflammatory mediators not only aids us in the follow-up of ongoing autoimmune processes, eventually full-blown autoimmune diseases, but also provides a tool to target and control these entities. Following inflammatory mediators reflecting autoimmunity helps us to intervene rapidly, before the eventual development of the disease and by doing so organ damage can be prevented. Knowledge and understanding of the pathogenic mechanisms that contribute to these conditions can lead to the development of novel diagnostic strategies and future effective therapies, providing better life expectancies to patients with autoimmune diseases.

In this special issue we present original research articles as well as review papers on the role of various inflammatory mediators in autoimmunity and autoimmune diseases.

The first review article from the editors gives an overview of the most important aspects in the pathogenesis of autoimmune diseases, with a special emphasis on the derailed balance between regulatory and Th17 cells. Additionally, we depict a cytokine imbalance, which gives rise to a biased T cell homeostasis in these patients. The review also portrays the multifaceted role of dendritic cells in the pathogenesis of autoimmunity; finally we describe the function and role of extracellular vesicles in particular autoimmune diseases.

The paper by N. Marton et al. describes the diverse roles of Src-like adaptor proteins (SLAP-1 and SLAP-2), their role in immunoregulation, and their effects on intracellular signaling pathways. The paper by C. Burbano et al. depicts the role of microparticles as modulatory structures in autoimmune processes and also introduces recent findings on microparticles in the pathogenesis in RA and SLE. The paper by S. G. Bourgoin et al. describes the effect of lysophosphatidic acid (LPA) on T cell recruitment via CXCL13 synthesis and its participation in inflammatory response regulation.

The development of lung fibrosis is a pathological process, characterized by abnormal accumulation of fibroblasts in the alveolar interstitium. The research article by P. Y. Cohen et al. depicts interesting aspects in the disease development in a mouse model by using gene chip analysis. The analysis concluded that lung myofibroblasts downregulated Thy1 expression and diminished the* in vivo* inflammatory milieu, indicating that inflammation is not essential for evolution of fibrosis. The paper by C. B. Holt el al. describes the role of Ficolin B in diabetic kidney diseases in a mouse model of type I diabetes and concludes that the molecule has no effect on diabetes-induced changes on the kidneys, opposed to the negative role of mannan-binding lectin in the pathogenesis. The next chapter of the special issue including 5 papers introduces various pathogenic aspects in systemic autoimmune and rheumatic diseases. In this unit, the articles introduce the clinical and immunoserological characteristics of rheumatic diseases with coinciding psoriasis, the role of progranulin in association with disease activity in RA patients, and a review article summarizes the role of poly (ADP-ribose) polymerase 1 in RA. A. A. S. S. K. Dharmapatni et al. in their paper describe the beneficial effects of Embelin, a XIAP inhibitor in a mouse model of collagen-induced arthritis, indicated by suppressed inflammation and reduced levels of the systemic bone resorption marker, CTX-1. Finally, in this section patients with ankylosing spondylitis were assessed and found that serum IL-6 correlated with ESR and CRP levels, making IL-6 a potentially important biomarker in the disease.

In this special issue we also depict various aspects in the pathogenesis of inflammatory bowel diseases (IBD). The paper by B. R. R. de Mattos et al. summarizes the latest findings in the immunological pathomechanism of IBDs and depicts the role of biologicals in the treatment while the paper by L. M. Medrano et al. describes that the analysis of a particular set of genes is a useful tool in the assessment of response to infliximab in Crohn's disease.

Follicular helper T cells (TFH) have been described to play a pivotal role in the initiation and perturbation of autoimmune processes leading to the development of autoimmune diseases. The paper by K. Szabo et al. suggests that in Sjögren's syndrome the presence of TFH cells in labial salivary gland biopsies at the disease onset is a good biomarker and may predict a more pronounced clinical course of the disease. The paper by X. Fan et al. describes the pathogenic role of TFH cells in human neuroautoimmune diseases and portrays their animal models.

This issue also illustrates the usefulness of inflammatory mediators in ocular manifestations of autoimmune diseases. The paper by A. Rentka et al. describes decreased levels of vascular endothelial growth factor (VEGF) in tear samples of patients with systemic sclerosis, while the paper by S. Sharma et al. reports from a study using a multiplex immunoassay that elevated serum levels of soluble TNF receptors and adhesion molecules are associated with diabetic retinopathy in patients with type-1 diabetes.

This special issue encompasses basic, molecular mechanisms of the pathogenesis in connection with autoimmune processes and autoimmune diseases, illustrating useful cellular and molecular mediators of inflammation. We believe that these data may contribute to improved tests for diagnosis and improve our knowledge of the underlying disturbances in the immune system in these hitherto unexplained disorders and hopefully these mediators of inflammation will be useful therapeutic targets in the future management of autoimmune diseases.

*Britt Nakken* *Britt Nakken* *György Nagy* *György Nagy* *Peter C. Huszthy* *Peter C. Huszthy* *Even Fossum* *Even Fossum* *Yrjö Konttinen* *Yrjö Konttinen* *Peter Szodoray* *Peter Szodoray*

